# Supplementary material for: Urushiol-Based Benzoxazine Containing Sulfobetaine Groups for Sustainable Marine Antifouling Applications
Source: Polymers (Basel). 2023 May 19;15(10):2383. doi: 10.3390/polym15102383 (PMC10221719; doi:10.3390/polym15102383)
Supplement: Supplementary file 1 [file polymers-15-02383-s001.zip › polymers-2385097-supplementary.pdf]

Electronic Supplementary Information

S1. Experimental

S1.1. Antiprotein adhesion assessments

The protein adhesion assay was adapted from the protocol already reported by Yang et al. [35]. The Micro BCA™ protein assay was used to measure the adsorption of BSA on the samples. BG, poly(U–ea), and poly(U–ea/sb) coating samples (2 cm × 2 cm) were first sterilized under UV light for 30 minutes and then equilibrated in sterilized PBS (pH = 7.4) for 2 hours, after which the samples were immersed in a protein solution at a concentration of 2 mg/mL. After incubation for 4 hours at 37°C, samples were gently washed 3 times with PBS (pH = 7.4) and then placed in 4 mL of PBS (pH = 7.4) containing 2 wt% sodium dodecyl sulphate (SDS) and sonicated in SDS solution for 30 minutes to remove BSA that did not adhere to the surface. Absorbance at 280 nm was obtained by measuring the absorbance at 280 nm using a UV/visible spectrophotometer. The concentration of BSA adhering to the surface of the coating was obtained by measuring the absorbance at 280 nm using a UV/vis spectrophotometer. γ-globulin was tested similarly.

S1.2. Antibacterial assessments

The antibacterial performance of poly(U–ea/sb) coatings was evaluated using four typical bacteria: Gram-negative *E. coli BW 25113* and *V. alginolyticus ATCC 33,787* and Gram-positive *S. aureus ATCC 25923* and *Bacillus sp. MCCC 1B00342*. The freezing stocks of bacterial strains were maintained at −80 ℃ in a 1:1 solution of Luria-Bertani (LB) broth: 40% (v/v) glycerol. Prior to use in antibacterial tests, the bacterial strains (*E. coli* and *S. aureus*) were first cultured in fresh LB broth at 37 ℃ by shaking at 170 rpm for 20 h until the $O.D._{600nm}$ of 1.8–2.0 was reached. Marine bacteria (*V. alginolyticus* and *Bacillus sp.*) were then incubated at 30°C. Then, BG, poly(U–ea), and poly(U–ea/sb) plates (2 cm × 2 cm) were cleaned by wiping with anhydrous ethyl alcohol, sterilized with ultraviolet radiation for 30 min, and placed in plastic Petri dishes [36-37]. The suspensions of bacteria were diluted to ca. 10^5~10^6 CFU/mL in fresh LB broth [38-40] and determined using a hemocytometer (Shanghai Qiujing Biochemical Reagent Instrument Co. Ltd., China). Subsequently, BG, poly(U–ea), poly(U–ea/sb) plates were inoculated with 100 μL of diluted bacterial suspension and covered with plastic wraps. The bacterial strains were incubated at 37℃ for 24 h under static conditions. (Marine bacteria are then incubated at 30℃). Afterwards, the plastic wraps were removed, and the plates were gently rinsed with 10 mL of sterile phosphate-buffered saline (PBS) to ensure that non-adhered bacteria were washed away. The antibacterial ratio (A. R.) of poly(U–ea/sb) coatings was quantified for the PBS rinse solution by counting the number of colonies present on the agar plate according to Eq. [4].

$$A.R. = \frac{N_{control} - N_{sample}}{N_{control}} \times 100\% \quad \text{(s1)}$$

where A.R. is the antibacterial ratio of UBMPs (%), $N_{control}$ is the number of bacterial colonies on BG plates (CFU/mL), and N_{sample} is the number of bacterial colonies on poly(U–ea) and poly(U–ea/sb) plates (CFU/mL). Each sample was measured three times to obtain the averaged value and standard deviations.

To quantify the amount of surface-adhered bacteria, the washed BG, poly(U–ea), and poly(U–ea/sb) plates were examined by FE-SEM (Hitachi SU8010, Japan). The PBS-rinsed samples were fixed in 2.5% glutaraldehyde, dehydrated in various concentrations of ethanol, freeze-dried, and then sprayed with gold. The samples were freeze-dried and then gold sprayed. FE-SEM was used to observe the surface morphology of the coating and the adhesion of bacteria to the surface.

S1.3 Algal biofouling assessments

To evaluate the effects of poly(U–ea/sb) coatings on algae, algal growth and attachment experiments were conducted. Algal cells *N. closterium*, *P. tricornutum*, and *D. zhan-*

jiangensis were grown in f/2 culture media, which were prepared in ASW at 22 ± 2 ℃ under a cycle of 12 h of fluorescent light and 12 h of dark. After 7 days of growth, the culture media containing algal cells were diluted with fresh culture media to give the test media concentrations of algal cells of 10^5–10^6/mL, which were used for the following algal attachment experiments. Similar to the antibacterial assessments, the sterilized BG, poly(U−ea), and poly(U−ea/sb) plates were immersed in a glass Petri dish containing 30 mL of culture media inoculated with *N. closterium* cells. After immersing for 1 day and 7 days, the concentrations of *N. closterium* cells were determined by counting the number of cells using a hemocytometer (Shanghai Qiujing Biochemical Reagent Instrument Co. Ltd., China), and optical photographs of the algal growth process were recorded. The same immersion process was applied to *P. tricornutum* and *D. zhanjiangensis* cells. After settling down, BG, poly(U−ea), and poly(U−ea/sb) plates were taken out from the test media and rinsed with 20 mL of sterile PBS to wash away any non-adhered algae. Subsequently, BG, poly(U−ea), and poly(U−ea/sb) plates were examined using a fluorescence microscope (Axio Imager A2, Zeiss, Germany), and the images of five random fields (40 × magnification, 0.156 mm^2/per field) were recorded for each sample. The algal coverage over BG, poly(U−ea), and poly(U−ea/sb) plates was determined by analyzing the fluorescence microscope images using the ImageJ software. All the results and standard deviations are based on three parallel experiments.

S1.4. ^{1}H-NMR testing of urushiol-based benzoxazine (U−ea) and urushiol (U) monomers

To determine the structure of the synthesized urushiol-based benzoxazine monomer U−ea, ^{1}H-NMR tests were performed on the monomer U−ea (using deuterated chloroform as solvent), as shown in Figure S1. In the ^{1}H-NMR spectra of the urushiol-based benzoxazine monomer (U−ea) and the urushiol (U), the solvent peak appeared at 7.28 ppm. Compared to the urushiol monomer, the U−ea monomer showed a peak at 4.86 ppm for the nuclear magnetic vibrations of the hydrogen atom on the oxazine ring O–CH_2–N and at 4.00 ppm for the Ar−CH_2−N. The ^{1}H-NMR also indicated the hydroxyl proton attached to the methylol group –CH_2O–H at 4.36 ppm. In addition, the multiple small peaks in the range of 3.48 ppm–4.25 ppm of the U−ea monomer spectrum may be due to the ring opening of some of the monomers to form dimers or multimers.

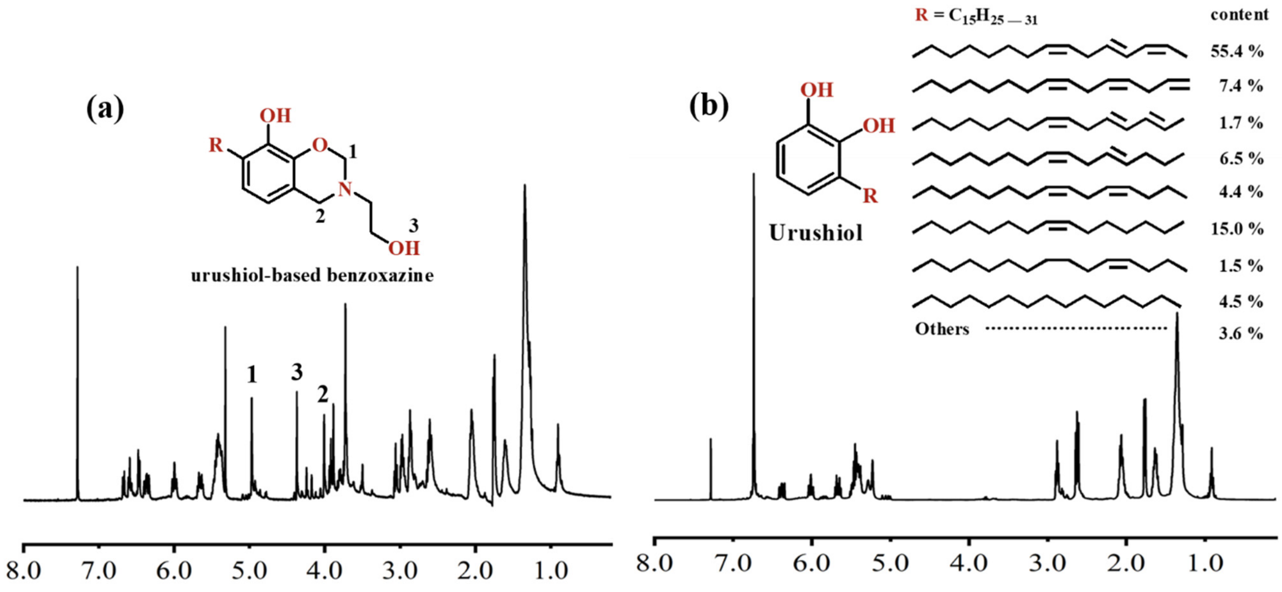

Figure S1. Quantitative analysis of the ^{1}H-NMR spectra of U and U−ea.

S2. Comparison of antifouling properties

In the present study, we utilized the modifiable molecular structure of the natural product urushiol and the exceptional antibacterial properties of zwitterion groups to produce an environmentally friendly, hydrophilic, and bio-based antifouling coating. This

coating is not only resistant to protein adsorption but also possesses antibacterial activity. In contrast, we found that only a limited number of antifouling coatings possess broad-spectrum antibacterial properties as well as effective antialgal properties. Table S1 presents a comprehensive comparison between the antibacterial and algal suppression capabilities of our coating and those of the referenced studies.

Table S1. Comparison of the functional properties of our poly(U−ea/sb) antifouling coatings and other works.

**WCA（°）**	**Typical polymers**	**Tested Fouling Materials**			Reference
		**Antibacterial**		Algal suppression	
		**Gram-positive**	Gram-negative		
**37.6°**	**Zwitterionic urushiol benzoxazine**	**>99.99%**	>99.99%	>99%	**This work**
×	PDA/AlgCAP@CSn	79%	74%	×	[41]
×	Bio-based amphiphilic epoxy/AgNPs	<60%	<87%	<99%	[42]
>79°	Ag@TA-SiO	82.7%	99%	<98%	[43]
>100°	QAS-SiO_2	91%~97%		54%~80%	[44]
~108°	PMMA@CuO/CuO-CTAB/ZnO	97%~99%		24%~38%	[45]
48.6°	Ca^{2+}-ISE@GO	53.1%		×	[46]
83°	Quaternary ammonium @GO	85%	90%	53%~84%	[47]
>60°	PBABs@PTPB	81.1%	95.1%	<92.8%	[48]

References

35. Yang, C.; Ding, X.; Ono, R.J.; Lee, H.; Hsu, L.Y.; Tong, Y.W.; Hedrick, J.; Yang, Y.Y. Brush-Like Polycarbonates Containing Dopamine, Cations, and PEG Providing a Broad-Spectrum, Antibacterial, and Antifouling Surface via One-Step Coating. *Advanced Materials.* **2014**, *26*, 7346-7351. doi:10.1002/adma.201402059.
36. Kacprzynska-Golacka, J.; Kowalik-Klimczak, A.; Skowronski, J.; Rajewska, P.; Wiecinski, P.; Smolik, J. Possibilities of using plasma techniques of surface engineering for modification of polymer membranes. *Polimery* .**2018**, *63*, 353-361. doi:10.14314/polimery.2018.5.4.
37. Kowalik-Klimczak, A.; Stanislawek, E. Reclamation of water from dairy wastewater using polymeric nanofiltration membranes. *Desalination and Water Treatment* **2018**, *128*, 364-371. doi:10.5004/dwt.2018.22981.
38. Sztuk - Sikorska, E.; Gradon, L. Biofouling reduction for improvement of depth water filtration. Filter production and testing. *Chemical and Process Engineering* **2016**, *37*, 319-330. doi:10.1515/cpe-2016-0026.
39. Kowalik-Klimczak, A.; Stanislawek, E.; Kacprzynska-Golacka, J.; Bednarska, A.; Osuch-Slomka, E.; Skowronski, J. The polyamide membranes functionalized by nanoparticles for biofouling control. *Desalination and Water Treatment* **2018**, *128*, 243-252. doi:10.5004/dwt.2018.22868.
40. Kacprzynska-Golacka, J.; Lozynska, M.; Barszcz, W.; Sowa, S.; Wiecinski, P.; Woskowicz, E. Microfiltration Membranes Modified with Composition of Titanium Oxide and Silver Oxide by Magnetron Sputtering. *Polymers (Basel)* **2020**, *13*, 141. doi:10.3390/polym13010141.
41. Hao, X.; Wang, W.; Yang, Z.; Yue, L.; Sun, H.; Wang, H.; Guo, Z.; Cheng, F.; Chen, S. pH responsive antifouling and antibacterial multilayer films with Self-healing performance. *Chemical Engineering Journal* **2019**, *356*, 130-141. doi:10.1016/j.cej.2018.08.181.
42. Lu, G.; Tian, S.; Li, J.; Xu, Y.; Liu, S.; Pu, J. Fabrication of bio-based amphiphilic hydrogel coating with excellent antifouling and mechanical properties. *Chemical Engineering Journal* **2021**, *409*. doi:10.1016/j.cej.2020.128134.
43. Deng, Y.; Song, G.-L.; Zheng, D.; Zhang, Y. Fabrication and synergistic antibacterial and antifouling effect of an organic/inorganic hybrid coating embedded with nanocomposite Ag@TA-SiO particles. *Colloids and Surfaces A: Physicochemical and Engineering Aspects* **2021**, *613*. doi:10.1016/j.colsurfa.2020.126085.
44. Wang, D.; Xu, J.; Yang, J.; Zhou, S. Preparation and synergistic antifouling effect of self-renewable coatings containing quaternary ammonium-functionalized SiO_2 nanoparticles. *J Colloid Interface Sci* **2020**, *563*, 261-271. doi:10.1016/j.jcis.2019.12.086.
45. Sathya, S.; Murthy, P.S.; Das, A.; Gomathi Sankar, G.; Venkatnarayanan, S.; Pandian, R.; Sathyaseelan, V.S.; Pandiyan, V.; Doble, M.; Venugopalan, V.P. Marine antifouling property of PMMA nanocomposite films: Results of laboratory and field assessment. *International Biodeterioration & Biodegradation* **2016**, *114*, 57-66. doi:10.1016/j.ibiod.2016.05.026.
46. Jiang, T.; Qi, L.; Qin, W. Improving the Environmental Compatibility of Marine Sensors by Surface Functionalization with Graphene Oxide. *Anal Chem* **2019**, *91*, 13268-13274. doi:10.1021/acs.analchem.9b03974.

47. Sha, J.; Chen, R.; Yu, J.; Liu, Q.; Liu, J.; Zhu, J.; Liu, P.; Li, R.; Wang, J. Dynamic multi-level microstructured antifouling surfaces by combining quaternary ammonium modified GO with self-polishing copolymers. *Carbon* **2023**, *201*, 1038-1047. doi:10.1016/j.carbon.2022.10.016.
48. Song, F.; Wang, J.; Zhang, L.; Chen, R.; Liu, Q.; Liu, J.; Yu, J.; Liu, P.; Duan, J. Synergistically Improved Antifouling Efficiency of a Bioinspired Self-renewing Interface via a Borneol/Boron Acrylate Polymer. *J Colloid Interface Sci* **2022**, *612*, 459-466. doi:10.1016/j.jcis.2021.12.187.
